# DAAF: Dual-stream adaptive attention fusion with distribution alignment for remote sensing object detection

**DOI:** 10.1371/journal.pone.0353309

**Published:** 2026-08-27

**Authors:** Mo Zhou, Yue Zhou, Kai Song

**Affiliations:** 1 School of Information Science and Engineering, Shenyang Ligong University, Shenyang, Liaoning, China; 2 School of Big Data, Liaoning Finance and Trade College, Huludao, Liaoning, China; Sichuan University, CHINA

## Abstract

Remote sensing object detection faces three challenges: extreme scale variation, arbitrary rotational orientations, and complex intermingled backgrounds. Although fusion of Convolutional Neural Networks (CNNs) and Transformers combines spatial precision with global modeling, it faces two limitations: (i) feature distribution misalignment between modalities, and (ii) ineffective exploitation of complementary discriminative strengths. To address these issues, we introduce DAAF (Dual-stream Adaptive Attention Fusion), a three-stage fusion framework that systematically aligns and integrates CNN and Transformer features. DAAF comprises three components: (1) The Unified Feature Distribution Calibration (UFDC) module applies instance normalization to align feature distributions, reducing Kullback-Leibler (KL) divergence by 94%; (2) The Heterogeneous Channel-wise Synergistic Selection (HCSS) module employs independent attention pathways for each stream, validated by zero overlap in top-10 channel weights between CNN and Transformer branches; (3) The Spatially Adaptive Discriminative-Preserving Gate (SADPG) module employs pixel-wise gating to adaptively balance fused and single-branch features. Experiments on three benchmarks (NWPU VHR-10, DOTA v1.0, DIOR) show consistent improvements averaging +2.05 percentage points (pp) in mean Average Precision (mAP) over the Add Fusion baseline, with substantial gains on structurally complex categories in NWPU (Bridge: + 17.36pp, Vehicle: + 13.18pp). These improvements are achieved with only 206K additional parameters (0.37% increase over baseline) and 7.2% latency rise compared to the baseline fusion method, demonstrating favorable efficiency for practical deployment.

## Introduction

**Fig 1:**
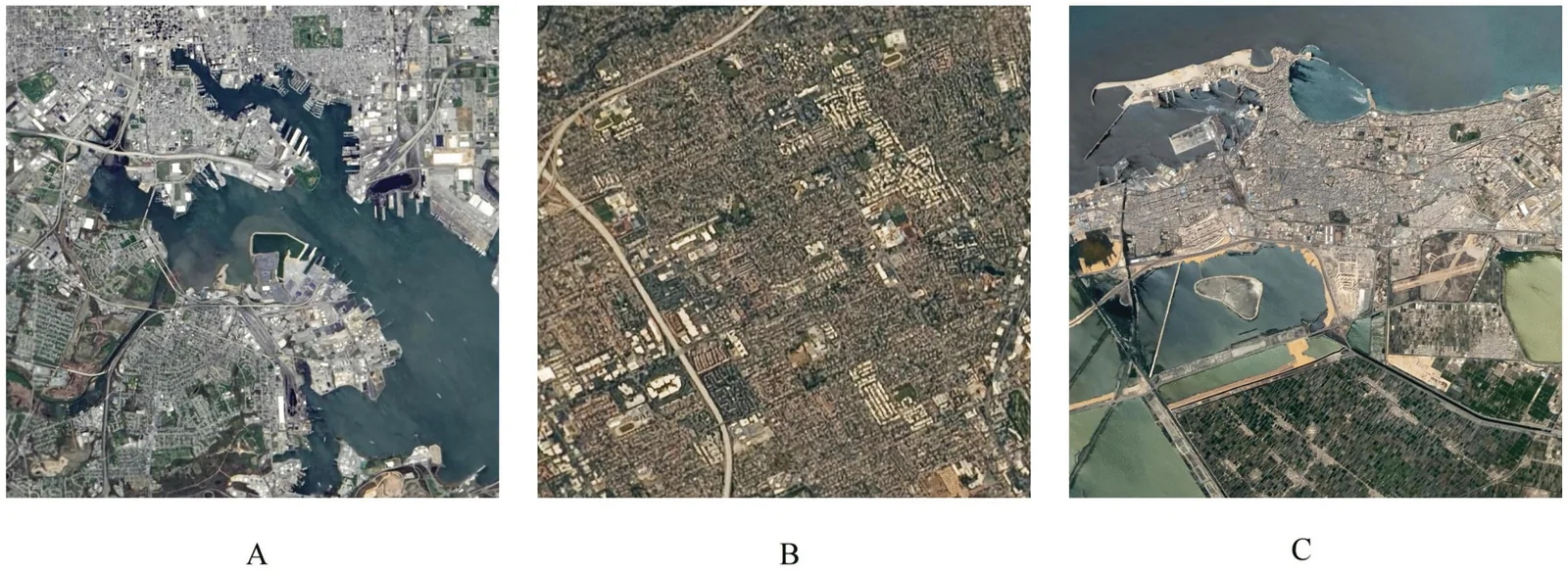
Representative remote sensing scenarios illustrating major challenges in object detection. (A) Complex backgrounds with heterogeneous land-cover patterns. (B) Small targets with limited discriminative information. (C) Objects exhibiting diverse spatial scales. The images were obtained from NASA Earth Observatory and were cropped and arranged by the authors for scientific illustration. NASA Earth Observatory is acknowledged as the original source, and the images were used in accordance with the applicable NASA image usage guidelines.

Remote sensing object detection, a core computer vision task essential for intelligent remote sensing image analysis, continues to gain growing significance across diverse application domains [1–3], such as disaster monitoring and emergency response [4,5], and urban and infrastructure monitoring [6–8]. The swift advancement of satellite and unmanned aerial vehicle (UAV) platforms has significantly expanded the availability and diversity of remote sensing imagery; meanwhile, the resolution and coverage of such images have been continuously improving, enabling automated object detection. Nevertheless, remote sensing imagery poses distinctive challenges for object detection—stemming from fundamental differences in imaging physics and geometric properties—compared with conventional natural image detection tasks. These challenges include extreme scale variations (e.g., from 10-pixel vehicles to 1000-pixel airports), arbitrary orientation rotations (spanning 0° to 360°), dense instance distributions (e.g., port areas containing hundreds of ships), and complex, heterogeneous background environments (such as urban areas, farmlands, and oceans). Fig 1 illustrates representative remote sensing scenarios and their associated challenges. These characteristics require the detection architecture to possess both fine-grained local detail capture capabilities and global context modeling capabilities simultaneously, which has become the core bottleneck restricting the performance improvement of remote sensing object detection.

Advances in deep learning have opened up novel methodological pathways for remote sensing object detection. Detectors based on CNNs, including Faster R-CNN [9], You Only Look Once (YOLO) [10], and RetinaNet [11], have demonstrated outstanding performance in both accuracy and inference speed, leveraging hierarchical convolutional operations to capture local patterns. In recent years, Vision Transformer [12] has demonstrated outstanding capabilities in global context modeling with its self-attention mechanism, bringing new opportunities to remote sensing detection. CNNs and Transformers offer complementary inductive biases for visual recognition. This natural complementarity has prompted researchers to explore dual-branch CNN-Transformer architectures for remote sensing detection.

However, effective CNN-Transformer fusion faces three critical technical obstacles: (1) **Feature Heterogeneity and Distribution Misalignment.** Remote sensing imagery is frequently degraded by atmospheric effects, variable acquisition conditions, and intricate scene compositions—leading to substantial background noise, pronounced visual similarity across object categories, and frequent occlusion of targets by structures, cloud cover, or shadow regions [13]. Although CNN detectors excel at extracting local detail features, they lack understanding of large-scale context relationships [14]. While introducing Transformer branches captures global information, the inherent statistical divergence between CNN and Transformer feature distributions hinders effective fusion. Without proper distribution alignment, feature conflicts arise, resulting in small target suppression in complex scenes, increased false positives/negatives, and imprecise boundary localization [15–17]. (2) **Multi-Scale Representation and Small Object Degradation.** Remote sensing imagery exhibits an extremely wide target scale spectrum, with densely distributed small objects often occupying only a handful of pixels and carrying limited semantic information [18]. In dual-branch architectures, repeated downsampling causes progressive degradation of fine-grained details in CNN branches, while Transformer branches produce overly smoothed global features where small targets exert minimal influence on attention maps. This multi-scale representation gap leads to severe feature loss for small objects and degradation of slender structures and boundaries. To address this, many approaches introduce multi-scale Transformers, feature pyramids, or hierarchical fusion mechanisms [19–21]. However, these solutions require cross-scale feature alignment at multiple pyramid levels, demanding additional projection modules at each fusion stage to maintain dimensional consistency—resulting in substantial growth in both parameter count and computational load, thereby imposing considerable burdens on model training and real-world deployment [19,22]. (3) **Fusion Architecture Complexity and Computational Overhead.** Due to the frequent interaction between dual branches at multiple scales and stages, additional fusion modules (attention mechanisms, pyramids, iterative fusions, etc.) need to be designed, resulting in channel transformations, attention calculations, or residual fusions at each scale, substantially raising computational complexity and model size [15,19]. Since Transformer branches inherently contain multi-head self-attention with wide channel dimensions, dimension alignment operations (e.g., 1×1 convolutions, linear projections, feed-forward networks) are often required for CNN and Transformer features during fusion. These projection layers and feed-forward networks multiply the number of parameters and matrix multiplications at each fusion point, especially on high-resolution remote sensing feature maps, which is more time-consuming [19,23].

To tackle the aforementioned challenges, we introduce DAAF (Dual-stream Adaptive Attention Fusion)—a novel fusion framework designed to boost detection accuracy for intricate objects by explicitly addressing feature distribution misalignment and underutilized channel complementarity in CNN-Transformer hybrid representations.

### Key contributions

This improvement is attributed to the following design elements:

(1) Dual-branch multi-scale feature extraction architecture. We design a backbone combining CNN (MobileNetV2) and Vision Transformer (MobileViT) branches to extract complementary local and global features at three Feature Pyramid Network levels (*P*_3_, *P*_4_, *P*_5_)—corresponding to downsampling strides of 8, 16, and 32 respectively (where subscripts denote 2^3^, 2^4^, 2^5^). This multi-level design comprehensively supports object detection across small, medium, and large instances. A channel-wise pre-alignment strategy ensures dimensional consistency between branches at each pyramid level, eliminating the need for projection layers and avoiding parameter increases. At each level, we apply our proposed three-stage fusion strategy—comprising UFDC, HCSS, and SADPG modules—to systematically integrate heterogeneous features.
(2) Unified Feature Distribution Calibration (UFDC). Instance normalization independently aligns CNN and Transformer feature distributions, eliminating numerical scale differences between high-variance CNN features and low-variance Transformer features. This prevents local activations from dominating fusion and enables effective utilization of global semantic information.
(3) Heterogeneous Channel-wise Synergistic Selection (HCSS). Independent attention pathways for CNN and Transformer branches learn channel importance weights, with Softmax normalization enforcing complementary selection. Channel attention analysis confirms zero overlap in top-10 channel weights between branches, validating the complementary nature of dual-stream features.
(4) Spatially Adaptive Discriminative-Preserving Gate (SADPG). A pixel-wise gating mechanism dynamically balances fused features with original CNN features through residual connections. This design ensures discriminative information is preserved during multi-level fusion, providing a performance lower bound even when fusion may not be optimal for certain spatial regions.

Together, these components constitute the DAAF (Dual-stream Adaptive Attention Fusion) framework for remote sensing object detection.

The rest of this paper is organized as follows: the Related Work section reviews the related work on remote sensing object detection, CNN-Transformer architectures, and feature fusion strategies; the Method section elaborates in detail on the methodology of DAAF; the Experiments and Results section presents comprehensive experimental evaluations and detailed comparative analyses across three benchmark datasets; the Discussion, Conclusion and Future Work section provides a comprehensive summary.

## Related work

### Remote sensing detection methods

In recent years, remote sensing object detection technology has undergone a rapid evolution from pure CNN to pure Transformer. These two approaches exhibit distinct strengths and trade-offs across key dimensions—including multi-scale representation capability, sensitivity to small objects, and inference efficiency.

Conventional remote sensing object detection approaches predominantly rely on convolutional neural networks, leveraging multi-scale feature aggregation and contextual scene understanding to handle the inherent complexity of remote sensing imagery. Early works such as Hierarchical Robust CNN [24] enhanced the robustness to rotation and scale changes by integrating multi-scale convolutional features with multi-layer fully connected features. The Scene-Context Feature Pyramid Network (SCFPN) [25] explicitly models the “object-scene” relationship within the Faster R-CNN structure and uses a feature pyramid to handle multi-scale issues. With the development of the YOLO series, researchers began to explore improvements in few-shot learning and small object detection. For instance, few-shot remote sensing detection based on YOLOv3 [26] introduced meta-learning and multi-scale detection heads while maintaining the end-to-end CNN structure to address the issue of scarce annotations. Recently, YOLO Squeeze-and-Excitation (YOLO-SE) [27] introduced lightweight convolutions, multi-scale attention modules, and additional small object detection heads on YOLOv8, further enhancing the detection performance of small objects. However, CNN-based methods are limited by local receptive fields, making it challenging to capture long-range spatial dependencies and holistic scene context—thereby degrading detection performance in complex, occluded, or densely populated remote sensing scenes.

Following the widespread success of Vision Transformers (ViTs) in computer vision, fully Transformer-based object detectors have emerged and advanced rapidly within remote sensing applications. The remote sensing detector based on Deformable Detection Transformer (DETR) [28] achieves end-to-end detection without Non-Maximum Suppression (NMS) and anchor boxes by fully using the Transformer encoder-decoder for feature modeling and prediction through dynamic anchor point queries, multi-pattern dynamic anchor object queries, and graph convolution priors. OrientedFormer [29] extends the DETR paradigm to rotated bounding box detection by introducing Gaussian positional encoding, geometric Wasserstein self-attention, and Transformer-based cross-attention—thereby enabling end-to-end orientation-aware detection without hand-crafted post-processing. In terms of the detection framework with ViT as the backbone, EIA-pyramid vision Transformer (EIA-PVT) [30] proposes an efficient inductive Vision Transformer, using mechanisms such as sparse routing and angle tokenization to complete the detection of remote sensing rotated targets under a full Transformer backbone, focusing on solving the problems of computational complexity and direction modeling of pure Transformer in high-resolution remote sensing. Frequency Feature Refinement Vision Transformer (FFRViT) [31] refines the frequency domain features, performing frequency domain division and refinement of local, global, and contextual features at multiple stages. Optimized Data Distribution Learning Net(ODDL-Net) [32] demonstrates that ViT backbones can significantly outperform CNN-based baselines in remote sensing detection. For small-target detection, Sparse Transformer-based Sparse Transformer-based Detector (STDet) [33] improves small-target feature representation and sampling allocation within a local-to-global attention framework via Local-Global Transformer blocks (LGFormer).

Pure CNN methods excel in local feature extraction and computational efficiency, but lack the ability to model global context. Conversely, pure Transformer methods, while capable of capturing long-range dependencies through self-attention, suffer from three limitations: high computational costs, insensitivity to local details, and overly smooth features that degrade small target detection. Motivated by these complementary strengths and weaknesses, the research community has turned to hybrid dual-path designs that integrate convolutional and self-attention mechanisms—capitalizing on CNNs’ local feature extraction and Transformers’ global contextual modeling to boost detection accuracy in remote sensing imagery without significantly increasing computational overhead.

### CNN-transformer dual-branch method

In recent years, growing attention has been devoted to investigating the CNN-Transformer dual-branch architecture to address the limitations of pure CNN methods—namely, their inability to model global context—and pure Transformer methods, which suffer from high computational overhead and inadequate local feature fidelity. Designed to harness both fine-grained local patterns and holistic scene-level context, this architecture integrates CNN-derived and Transformer-derived features through either concurrent (parallel) or cascaded (sequential) fusion strategies.

The core idea of the dual-branch architecture is that CNNs excel at capturing localized texture and boundary information, whereas Transformers are adept at encoding global spatial relationships and semantic category awareness [34–36]. Typical architectural designs include: (1) a CNN backbone for extracting multi-scale local features; (2) a Transformer module for modeling global dependencies; (3) a feature fusion module to combine the complementary information from both. This paradigm has demonstrated significant advantages in remote sensing object detection.

Early dual-branch methods mostly adopted a serial fusion strategy, that is, first using CNN to extract features and then having Transformer perform global modeling. Transformer for Remote-Sensing Object Detection (TRD) and TRD with the Transferring CNN (T-TRD) [34] employs a CNN to extract multi-scale features and subsequently leverages a multi-layer Transformer encoder-decoder for global relationship modeling and set-based object prediction—thereby eliminating the need for hand-crafted anchors, non-maximum suppression (NMS), and Feature Pyramid Network (FPN). It outperformed YOLOv3 and Faster R-CNN + FPN in small-target detection. CNN and Multiscale Transformer Fusion Network (CMTFNet) [17] and CNN-Transformer Fusion Network (CTFNet) [37] followed a similar approach, first encoding features with CNN and then modeling multi-scale global context with the Transformer decoder (such as multi-scale Multi-Head Self-Attention (MHSA), lightweight Transformer block), and finally fusing with shallow CNN features through an attention module at the decoder end. The advantage of the serial architecture lies in its simplicity of implementation, allowing the direct “insertion” of Transformer modules into mature CNN detection frameworks. However, it has the problem of limited interaction between CNN and Transformer, and the complementary nature of local and global features is not fully exploited [38].

The parallel architecture explicitly builds CNN and Transformer branches, which are both forward-propagated simultaneously, and then the features are aligned and interacted through a dedicated fusion module. Methods such as CNN and Transformer Multiscale Fusion Network (CTMFNet) [39] and CNN-Transformer Complementary and Fusion Network(CTCFNet) [40] adopt a dual-encoder parallel design: a dedicated CNN branch captures fine-grained local features, while a complementary Transformer branch encodes global contextual representations; these two feature streams are then deliberately integrated across multiple scales using either dual-backbone attention mechanisms or hierarchical feature aggregation modules. Variants like W-shaped hierarchical Network (WNet) [41] achieve cross-branch and cross-scale information flow through multi-level CNN-Transformer fusion modules and full-scale skip connections. The advantage of the parallel architecture lies in that local textures, boundaries, and global semantics can interact deeply at the same scale and stage, which is more beneficial for handling small objects and complex backgrounds.

### Feature fusion methods

**Fig 2:**
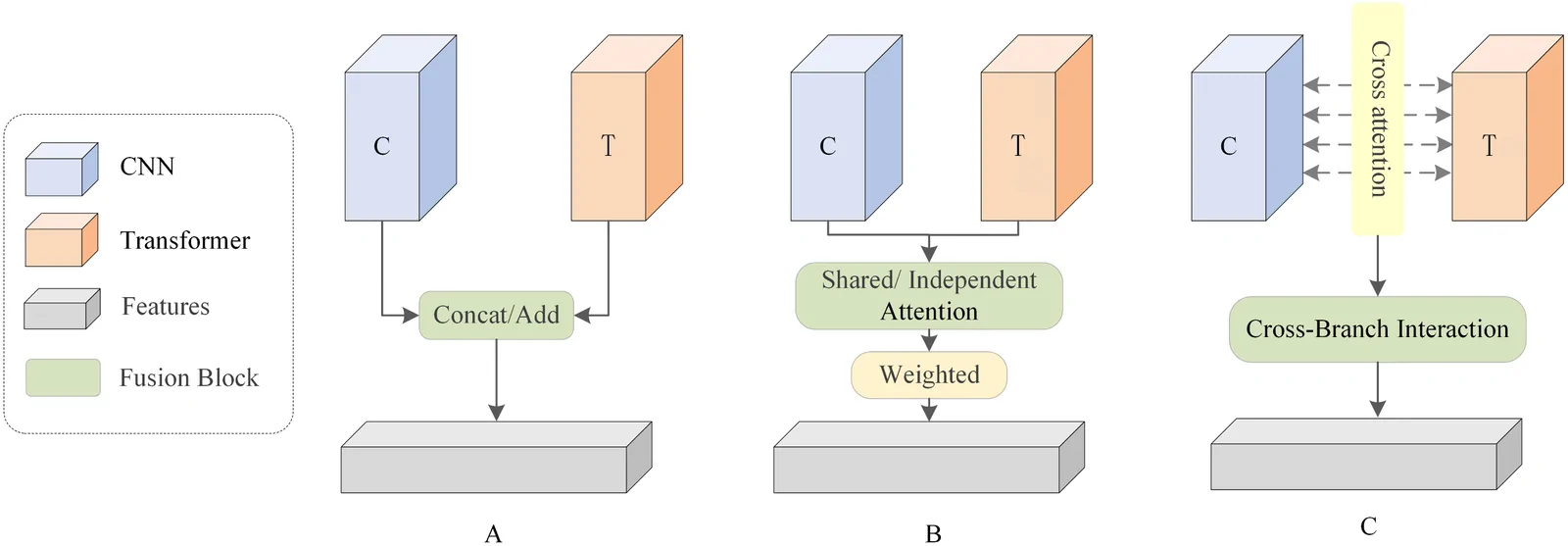
Structures of existing CNN-Transformer fusion methods. (A) Static arithmetic fusion; (B) Attention-weighted fusion; (C) Cross-branch interaction fusion.

Existing CNN-Transformer fusion methods can be categorized into three groups according to the increasing complexity of their fusion strategies, As shown in Fig 2.

In many CNN-Transformer dual-branch architectures, a common baseline fusion strategy is to first align the features of the two branches in the spatial and channel dimensions, then combine them through element-wise addition or channel concatenation, and use lightweight convolution for channel compression. Such methods are straightforward to implement and computationally efficient; nevertheless, these methods realize implicit branch alignment through the aforementioned fusion operations based on an ideal premise that branch features are well-aligned. Such a favorable condition is hard to satisfy in real-world tasks, and they rarely equip dedicated modules to explicitly model cross-branch channel importance and branch-level weighting relations. Therefore, effectively characterizing the heterogeneity and complementarity between CNN and Transformer representations remains an open design question [17,42].

Attention-weighted fusion [43] introduces learnable attention or gating mechanisms to assign weights to dual-branch features based on channel or spatial importance. Depending on the way weights are generated, this category of methods can be further divided into shared attention (generating unified weights from the fused features) and independent attention (generating weights separately and then adding them). Directly applying shared attention mechanisms like Convolutional Block Attention Module (CBAM)/SE [44] on the fused features amounts to generating a single set of channel weights for both the CNN and Transformer branches, implicitly treating their channel-importance distributions as similar. In contrast, some works design independent attention or gating for different modalities/branches to better exploit complementary information [43,45]. The shared-attention strategy does not explicitly distinguish complementary roles between CNN and ViT features, whereas independent attention usually lacks explicit regularization to encourage complementary behavior between branches.

The third approach involves cross-branch interaction-based feature fusion [46,47], where CNN and Transformer representations are dynamically integrated via a cross-attention mechanism, facilitating mutual enhancement in both directions. While cross-attention mechanisms enable powerful feature interaction, usually incur higher computational cost. Moreover, repeated cross-branch interaction may risk over-smoothing sparse discriminative features, especially in remote sensing scenes where targets typically occupy only a small portion of the image, and the effectiveness of interaction can be affected if feature distributions across branches are not well aligned.

## Methods

This section systematically elaborates the theoretical underpinnings and architectural implementation of DAAF (Dual-stream Adaptive Attention Fusion), detailing how its two-stream design dynamically modulates attention across modalities to achieve adaptive feature integration.

### Overall structure

We denote the input remote sensing image as a three-channel tensor $I\in {\mathbb{R}}^{H\times W\times 3}$, where *H* and *W* represent its height and width, respectively. We adopt a dual-branch architecture to extract features from CNN and Transformer, and fuse them at three levels of the feature pyramid ($P_{3}$, $P_{4}$, $P_{5}$). Specifically, the CNN branch (MobileNetV2 [48]) generates $F_{CNN}^{P_{3}}$, $F_{CNN}^{P_{4}}$ and $F_{CNN}^{P_{5}}$, while the ViT branch (MobileViT [49]) generates $F_{ViT}^{P_{3}}$, $F_{ViT}^{P_{4}}$ and $F_{ViT}^{P_{5}}$. Here, $F_{ViT}^{l},F_{CNN}^{l}\in {\mathbb{R}}^{C_{l}\times H_{l}\times W_{l}}$ represent the feature maps at pyramid level *l*, where $C_{l}\in \{128,256,256\}$ and $H_{l}\times W_{l}\in \{80\times 80,40\times 40,20\times 20\}$ correspond to $l\in \{P_{3},P_{4},P_{5}\}$, respectively. The subscripts 3, 4, and 5 denote progressively deeper pyramid levels in the backbone network, corresponding to feature maps with increasing downsampling factors relative to the input image. Following the standard Feature Pyramid Network (FPN) convention, pyramid levels used for detection start from $P_{3}$, since $P_{1}$ and $P_{2}$ mainly capture low-level visual details (e.g., edges and textures) with limited semantic information and therefore are generally not employed as prediction layers in object detection. The three *l*evels of the feature pyramid correspond to different semantic levels: the $P_{3}$ layer is responsible for detecting small objects, the $P_{4}$ layer processes medium-sized objects, and the $P_{5}$ layer focuses on large objects.

We choose a lightweight architecture to balance detection accuracy and computational efficiency. MobileNetV2 leverages depthwise separable convolutions to extract fine-grained local features—including edges and textural patterns—efficiently. In contrast, MobileViT adopts a hybrid CNN–Transformer design that jointly achieves lightweight deployment and robust global contextual understanding.

**Fig 3:**
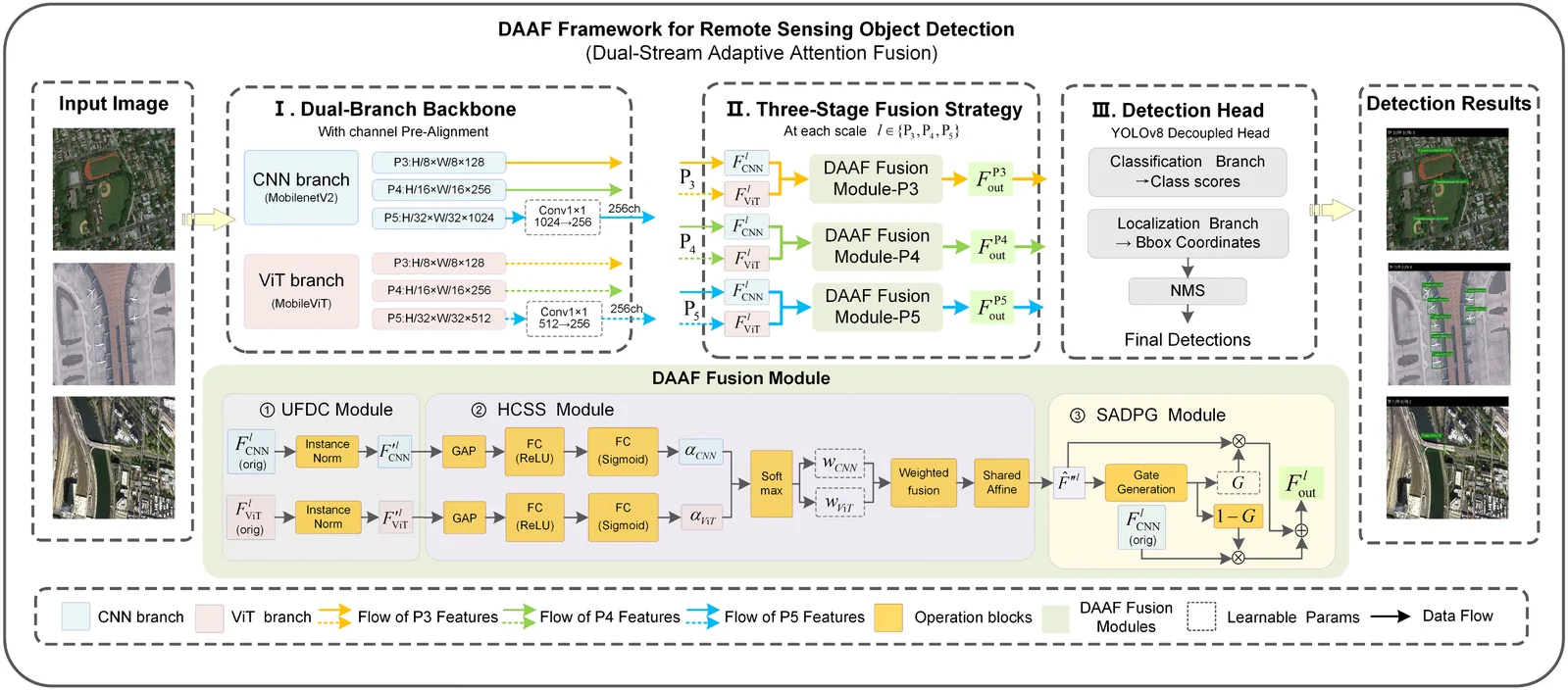
Overview of the DAAF architecture. The input image is passed through a dual-path backbone composed of MobileNetV2 and MobileViT, enabling hierarchical multi-scale feature extraction. The DAAF module is applied independently at each of the three FPN levels—$P_{3}$, $P_{4}$, and $P_{5}$. Each DAAF module operates in three sequential stages, and the resulting fused features are forwarded to the detection head for final prediction generation.

Fig 3 shows the overall architecture of DAAF. At each feature pyramid level $l\in \{P_{3},P_{4},P_{5}\}$, DAAF processes the features from the CNN and ViT branches in three collaborative stages in sequence. In the first stage—distribution alignment—instance normalization is applied to $F_{CNN}^{l}$ and $F_{ViT}^{l}$ independently, normalizing each to a zero-mean, unit-variance distribution; this eliminates inter-feature-scale discrepancies and establishes a consistent basis for subsequent fusion. In the second stage (complementary utilization), a dual-branch Channel Attention is employed to independently learn channel importance weights for the CNN and ViT, and Softmax normalization is used to enforce complementary weights, enabling the network to dynamically allocate fusion budgets based on the discriminative power of each channel. In the third stage (information protection), a spatially adaptive Residual Gate is introduced, which learns position-related fusion weights through 1×1 convolution, while protecting the key discriminative information in the original CNN features through residual connections while leveraging complementarity. The features fused by DAAF are then sent to the standard YOLOv8 decoupled detection head for final object localization and classification.

### Dual-branch feature extraction architecture

In designing a feature fusion network, the strategic placement of fusion nodes critically influences the model’s capacity to detect objects across diverse scales. In this work, three representative feature levels ($P_{3}$, $P_{4}$, $P_{5}$) are selected for cross-modal fusion based on three considerations: (1) Detector compatibility. modern detection frameworks (YOLOv8, Faster R-CNN) employ three-level Feature Pyramid Networks as the standard backbone-head interface, ensuring seamless integration without architectural modifications; (2) Channel alignment efficiency. as shown in Table 1, MobileNetV2 and MobileViT achieve natural channel alignment at $P_{3}$ (128 channels) and $P_{4}$ (256 channels) without modification, with only minimal adjustment at $P_{5}$ (1024→256, 512→256), minimizing projection overhead; (3) Semantic complementarity across scales. the three levels correspond to shallow, middle, and deep semantic information respectively, where CNN-Transformer complementarity manifests differently at each perception stage (detailed below). We adjusted the number of channels in the two branches to ensure that the feature maps output at the shallow, middle, and deep feature levels have 128, 256, 256 channels respectively.

**Table 1:** Channel Alignment Configuration for Dual-Branch Fusion.

Backbone Information			Channel Configuration			
Backbone	Fusion Level	Block Type	Detection Baseline (Original Channels)	DAAF Fusion (Aligned Channels)	Spatial Resolution	Output Channels
MobileNetV2	$P_{3}$	Inv Res	t=6,c=128,n=3	t=6,c=128,n=3	H/8×W/8	128
	$P_{4}$		t=6,c=256,n=3	t=6,c=256,n=3	H/16×W/16	256
	$P_{5}$		**t=6,c=1024,n=1**	**t=6,c=256,n=1**	** H/32×W/32 **	**1024/256**
MobileViT	$P_{3}$	MobileViTBlock	ch = 128	ch = 128	H/8×W/8	128
	$P_{4}$		ch = 256	ch = 256	H/16×W/16	256
	$P_{5}$	ConvBlock+MobileViT	**ch = 512**	**ch = 256**	** H/32×W/32 **	**512/256**

Note: InvRes = Inverted Residual block; t = expansion ratio; c = output channels; n = number of blocks; ch = channels.

Through the above customized design, the dual-branch network achieves precise scale alignment at three key levels:

$P_{3}$ alignment: Spatial resolution is H/8×W/8, and both branches output 128-channel feature maps.
$P_{4}$ alignment: Spatial resolution is H/16×W/16, and both branches output 256-channel feature maps.
$P_{5}$ alignment: Spatial resolution is H/32×W/32, and both branches output 256-channel feature maps.

As shown in Table 1, only the $P_{5}$ level requires channel adjustment: MobileNetV2 is reduced from 1024 to 256 channels, and MobileViT is reduced from 512 to 256 channels. The $P_{3}$ and $P_{4}$ levels do not need to be modified to achieve alignment, which minimizes the changes to the original backbone network, eliminates the overhead generated during the alignment of projection layers, and at the same time, the 256-channel configuration has been experimentally verified to be able to fully retain deep semantic information and maintain representational ability. The dual branches achieve dimensional consistency at each level, creating ideal conditions for feature interaction in the DAAF module and matching the input requirements of the detection head.

The core motivation for fusing three levels lies in leveraging the inherent complementary characteristics of CNN and Transformer architectures at different perception stages.

At the shallow layer ($P_{3}$), CNN operators excel at extracting fine local patterns and detailed features, while the lightweight Transformer module can already conduct preliminary modeling of semantic associations within local regions. The feature perspectives provided by the two at this level have significant differences and are highly complementary in information. This fusion step is designed to strengthen the model’s capacity to capture intricate image details, leading to more reliable detection of small objects.

At the middle and deep layers ($P_{4}$, $P_{5}$), as the receptive field expands, the high-level semantic information of CNN features continuously strengthens, while the Transformer further establishes long-range dependencies between image blocks, forming a global context understanding. The fusion at this stage aims to effectively inject the global structured information captured by the Transformer into the hierarchical features of the CNN, to make up for the CNN’s deficiency in modeling long-distance relationships, thereby optimizing the recognition and localization performance for medium and large-scale targets.

### Unified feature distribution calibration module (UFDC)

CNN and Transformer generate significantly different feature distributions due to their distinct architectures and inductive biases. CNN captures local texture and edge features through convolution operations, with its activation values mainly concentrated in high-gradient regions, presenting a high variance distribution; while ViT models global dependencies through self-attention mechanisms, with its activation values relatively smooth in space, presenting a low variance distribution. This distribution difference leads to a numerical scale mismatch during fusion: the low-variance ViT features are easily “dominated” numerically by the high-variance CNN features, resulting in the ineffective integration of ViT’s global semantic information (such as scene-level spatial relationship understanding) into the final features.

To eliminate the differences in numerical scales, we propose a unified feature distribution calibration module. By performing instance normalization and shared affine transformation on the features of the two branches, they are calibrated to a unified target distribution, thereby achieving balanced fusion of heterogeneous features in the numerical scale and providing a stable and consistent feature input for the subsequent detection head. Instance Normalization is used to independently normalize the CNN and ViT features. Given a feature tensor $F\in {\mathbb{R}}^{N\times C\times H\times W}$ (from the CNN or ViT branch), Instance Normalization computes the statistics and normalizes them independently for each sample s∈{1,...,N} and each channel c∈{1,...,C}. Specifically, for each sample *s* and each channel *c*, the mean $\mu_{s,c}$ and standard deviation $\sigma_{s,c}$ are first calculated over the spatial dimension (H×W):


$$\begin{array}{c} \mu_{s,c}=\frac{1}{HW}\sum_{h=1}^{H}\sum_{w=1}^{W}F(s,c,h,w) \end{array}$$ (1)

$$\begin{array}{c} \sigma_{s,c}=\sqrt{\frac{1}{HW}\sum_{h=1}^{H}\sum_{w=1}^{W}{\left(F(s,c,h,w)-\mu_{s,c}\right)}^{2}} \end{array}$$ (2)

Subsequently, feature standardization is applied to normalize the representations to a zero-mean, unit-variance distribution:


$$\begin{array}{c} F^{\prime }(s,c,h,w)=\frac{F(s,c,h,w)-\mu_{s,c}}{\sigma_{s,c}+\varepsilon } \end{array}$$ (3)

Here, $\varepsilon =10^{-5}$ is a small smoothing term introduced to avoid numerical instability caused by division by zero. Subsequently, the normalized features $F^{\prime }(s,c,h,w)$ from both branches are forwarded to the Heterogeneous Channel-wise Synergistic Selection (HCSS) module for adaptive channel-wise fusion.

### Heterogeneous channel-wise synergistic selection (HCSS)

Although Instance Normalization successfully aligns the global distribution of features, CNNs and ViTs still maintain significant complementarity at the channel level – this complementarity stems from the different inductive biases of the two architectures and does not disappear due to normalization. The convolutional operations of CNNs are naturally sensitive to local patterns (edges, textures, corners), while the self-attention mechanism of ViTs tends to aggregate global semantics (shapes, layouts, contextual relationships). This architectural difference leads to systematic differences in channel selection between the two branches.

Based on the unified feature distribution calibration, we further propose the heterogeneous channel collaborative selection module. This module equips the CNN and Transformer branches with independent attention paths, enabling each branch to learn the channel importance weights based on its own features. Moreover, the Softmax constraint ensures that the learned weights are inherently complementary, enabling adaptive and context-aware integration of local texture cues and global semantic information—ultimately yielding more discriminative, fused representations for the detection head.

Given the normalized features $F_{CNN}^{\prime }(s),F_{ViT}^{\prime }(s)\in {\mathbb{R}}^{C\times H\times W}$ for sample *s*, the spatial information is first compressed through global average pooling:


$$\begin{array}{c} z_{CNN}(s)=GAP(F_{CNN}^{\prime }(s))\in {\mathbb{R}}^{C} \end{array}$$ (4)

$$\begin{array}{c} z_{ViT}(s)=GAP(F_{ViT}^{\prime }(s))\in {\mathbb{R}}^{C} \end{array}$$ (5)

Subsequently, a two-layer fully connected network is employed to learn the channel-wise attention weights. For the CNN branch:


$$\begin{array}{c} \alpha_{CNN}(s)=\sigma ({FC}_{2}^{CNN}(ReLU({FC}_{1}^{CNN}(z_{CNN}(s))))) \end{array}$$ (6)

Here, ${FC}_{1}^{CNN}:{\mathbb{R}}^{C}\to {\mathbb{R}}^{C/r}$ and ${FC}_{2}^{CNN}:{\mathbb{R}}^{C/r}\to {\mathbb{R}}^{C}$ are two layers of fully connected networks for the CNN branch, *r* is the reduction ratio, and σ is the Sigmoid activation function. The ViT branch adopts the same structure but with completely independent parameters ${FC}_{1}^{ViT}$ and ${FC}_{2}^{ViT}$.


$$\begin{array}{c} \alpha_{ViT}(s)=\sigma ({FC}_{2}^{ViT}(ReLU({FC}_{1}^{ViT}(z_{ViT}(s))))) \end{array}$$ (7)

It should be emphasized that the FC layers (${FC}_{1}^{CNN}$, ${FC}_{2}^{CNN}$) and (${FC}_{1}^{ViT}$, ${FC}_{2}^{ViT}$) of the two branches are completely independent and do not share any parameters, which ensures that each branch can learn the channel selection mode suitable for its own features.

Softmax normalization is the key mechanism for achieving complementarity. To enforce the complementary weights of the two branches, we apply Softmax normalization to the original weights $\alpha_{CNN}$ and $\alpha_{ViT}$:


$$\begin{array}{c} w_{CNN}(s)=\frac{\alpha_{CNN}(s)}{\alpha_{CNN}(s)+\alpha_{ViT}(s)+\varepsilon } \end{array}$$ (8)

$$\begin{array}{c} w_{ViT}(s)=\frac{\alpha_{ViT}(s)}{\alpha_{CNN}(s)+\alpha_{ViT}(s)+\varepsilon } \end{array}$$ (9)

Here, $\varepsilon =10^{-8}$ is used to prevent division by zero. Through this normalization, we ensure that $w_{CNN}+w_{ViT}=1$ (channel-wise), which enforces complementary weights: when $w_{CNN}$ has a higher weight for channel *c*, $w_{ViT}$ must be lower, and vice versa. This design enables the network to naturally capture channel negative correlations – for channels that are important to CNN but not to ViT (such as edge features), the network tends to assign $w_{CNN}>w_{ViT}$; for channels that are important to ViT but not to CNN (such as global semantics), the network assigns $w_{ViT}>w_{CNN}$.

Weighted fusion and affine transformation. After obtaining the complementary channel weights, perform channel-wise weighted fusion:


$$\begin{array}{c} F^{\prime \prime }(s,c,h,w)=w_{CNN}(s,c)⊙F_{CNN}^{\prime }(s,c,h,w)+w_{ViT}(s,c)⊙F_{ViT}^{\prime }(s,c,h,w) \end{array}$$ (10)

Here, ⊙ denotes channel-wise multiplication (the weights $w_{CNN}(s,c)$ and $w_{ViT}(s,c)$ are broadcasted along the spatial dimensions).

Shared Affine Transformation. After obtaining the channel-weighted fusion $F^{\prime \prime }(s,c,h,w)$, we apply a shared affine transformation to restore the expressive power and align both branches to a unified target distribution:


$$\begin{array}{c} {\hat{F}}^{\prime \prime }(s,c,h,w)=\gamma_{c}\cdot F^{\prime \prime }(s,c,h,w)+\beta_{c} \end{array}$$ (11)

Here, $\gamma_{c},\beta_{c}\in \mathbb{R}$ are learnable scalar parameters for each channel *c*, shared across all samples and spatial positions. Importantly, we use the same $\gamma_{c}$ and $\beta_{c}$ for both the CNN and ViT branches, ensuring that the two branches are aligned to a unified target distribution while allowing the network to learn the optimal feature scale through gradient descent.

Finally, the affine-transformed features ${\hat{F}}^{\prime \prime }(s,c,h,w)$ are forwarded to the Spatially Adaptive Discriminative-Preserving Gate (SADPG) module for spatially adaptive gating.

### Spatially adaptive discriminative-preserving gate (SADPG)

Inspired by the learnable gating in Highway Networks [50], we introduce a spatially adaptive gating mechanism to control the fusion process, ensuring that the key discriminative information is not diluted during the fusion. This gating dynamically learns weights in the spatial dimension and adaptively weights the fused features and the original CNN features in a residual form, thereby intelligently balancing the complementary benefits and the information preservation requirements in complex scenarios.

Given the fused feature ${\hat{F}}^{\prime \prime }(s)\in {\mathbb{R}}^{C\times H\times W}$ after affine transformation, we learn position-dependent gating weights through a 1×1 convolution:


$$\begin{array}{c} G(s,c,h,w)=\sigma ({Conv}_{1\times 1}({\hat{F}}^{\prime \prime }(s))) \end{array}$$ (12)

Here, ${Conv}_{1\times 1}$ denotes a 1×1 convolutional layer featuring *C* input and *C* output channels, configured without a bias term to minimize parameter count; σ denotes the sigmoid activation function, ensuring that all gating signals are bounded within the range [0, 1].

Residual gated fusion is the core design for protecting discriminative information. Instead of directly using the fused features, we perform a weighted combination between the fused features and the original CNN features through gating:


$$\begin{array}{c} F_{out}(s,c,h,w)=G(s,c,h,w)⊙{\hat{F}}^{\prime \prime }(s,c,h,w)+(1-G(s,c,h,w))⊙F_{CNN}^{orig}(s,c,h,w) \end{array}$$ (13)

Here, ${\hat{F}}^{\prime \prime }(s)$ is the fused feature after UFDC and HCSS, which contains complementary information from CNN and ViT; $F_{CNN}^{orig}(s)$ is the output of the original CNN backbone (without any processing), retaining complete local discriminative features; ⊙ indicates element-wise multiplication.

The semantic meaning of the gating value *G* can be understood as follows: when *G* approaches 1, the network fully trusts the fused features, which is suitable for regions with good fusion effects (such as the main structure of the bridge category, where the CNN edges and ViT context complement each other strongly); when *G* approaches 0, the network reverts to the original CNN features, which is suitable for regions where fusion might lead to information loss (such as the weak edges of small objects, where the smoothing of ViT might weaken edge responses); when 0 < *G* < 1, the network adaptively blends the two, dynamically adjusting based on specific spatial positions and channel characteristics. More importantly, the residual design ensures that at least the original information of CNN is retained, and the fusion branch serves as an additive enhancement rather than a replacement. This theoretically provides a performance lower bound guarantee – even if the fusion fails, the performance will not be lower than that of using CNN alone.

At this point, we can obtain the detailed form of the final output:


$$\begin{array}{c} F_{out}=G⊙(\gamma (w_{CNN}⊙F_{CNN}^{\prime }+w_{ViT}⊙F_{ViT}^{\prime })+\beta )+(1-G)⊙F_{CNN}^{orig} \end{array}$$ (14)

This formula clearly shows the complete data flow of DAAF: the original features are normalized by UFDC ($F^{\prime }$), weighted and fused through the dual-branch HCSS ($w⊙F^{\prime }$), affine transformation is applied to restore the scale (γF+β), and finally dynamically mixed with the original CNN features through SADPG.

## Experiments and results

### Experimental setup and experimental details

#### Datasets.

We comprehensively evaluated the proposed DAAF framework through large-scale experiments on three widely adopted aerial remote sensing detection benchmarks: NWPU VHR-10 [51], DOTA v1.0 [52], and DIOR [53]. These three datasets cover different geographical regions, imaging conditions, and object categories, as shown in Table 2. Their complementary characteristics enable us to comprehensively verify the generalization ability and applicability boundaries of the method.

**Table 2:** Specific Categories and Codes of the Three Datasets.

Dataset	Specific Categories and Codes				
DIOR	C1	C2	C3	C4	C5
	Airplane	Airport	Baseball Field	Basketball Court	Bridge
	C6	C7	C8	C9	C10
	Chimney	Dam	Expressway service area	Expressway toll station	Golf Field
	C11	C12	C13	C14	C15
	Ground Track Field	Harbor	Overpass	Ship	Stadium
	C16	C17	C18	C19	C20
	Storage Tank	Tennis Court	Train Station	Vehicle	Windmill
DOTA	BD	BC	BR	GTF	HA
	Baseball Diamond	Basketball Court	Bridge	Ground Field Track	Harbor
	HC	LV	PL	RA	SH
	Helicopter	Large Vehicle	Plane	Roundabout	Ship
	SV	SBF	ST	SP	TC
	Small Vehicle	Soccer-ball Field	Storage Tank	Swimming Pool	Tennis Court
NWPU	AP	BD	BC	BR	GTF
	Airplane	Baseball-Diamond	Basketball-Court	Bridge	Ground-Track-Field
	HB	SH	ST	TC	VH
	Harbor	Ship	Storage-Tank	Tennis-Court	Vehicle

The NWPU VHR-10 dataset contains 800 high-resolution aerial remote sensing images, each labeled with instances belonging to ten predefined object classes. The dataset is characterized by a moderate scale, high category diversity, and substantial intra-class and inter-class appearance variations, making it especially suitable for analyzing performance differences across target categories with varying structural complexities.

The DOTA v1.0 dataset is a large-scale oriented object detection dataset, featuring 15 categories and 2,806 images. It is highly challenging due to extreme scale variations (object sizes ranging from 10x10 to 500x500 pixels), arbitrary orientations (0–360 degrees), and diverse scene types (including ports, airports, and urban areas).

The DIOR dataset emphasizes high scene diversity and category imbalance, consisting of 20 categories, 23,463 images (resolution: 800x800 pixels), and 192,472 annotated instances. It has significant inter-class similarities (e.g., different types of stadiums share similar rectangular shapes) and severe category imbalance (45,303 instances for the vehicle category vs. 2,513 instances for the chimney category), effectively evaluating the robustness of methods to common data distribution challenges in real-world applications.

All datasets used in this study, including NWPU VHR-10, DOTA v1.0, and DIOR, were accessed from publicly available dataset repositories and used solely as benchmark datasets for scientific evaluation, including model training, validation, testing, and quantitative evaluation. The original dataset images and annotations remain the property of their respective copyright holders. The authors do not claim ownership of these materials, and the original dataset files were not redistributed in this study. Appropriate citations to the corresponding dataset publications have been provided throughout the manuscript.

#### Implementation details.

**Detector Architecture:** We implement DAAF on top of the YOLOv8-S [54] detection framework. Input images are uniformly scaled to 640×640 pixels.

**Training setup:** The model is optimized end-to-end over 150 training epochs with a mini-batch size of 8. We use Stochastic Gradient Descent (SGD) with momentum 0.937 and weight decay 5e-4. The learning rate starts at 0.01 and decays to $1\times 10^{-4}$ following a cosine annealing schedule. Mosaic and Mixup augmentations are applied with 0.5 probability during the first 105 epochs (70% of training), then deactivated to promote stable convergence. No label smoothing is used.

**Hardware and software environment:** All experiments were conducted on a single NVIDIA RTX 3090 GPU (24 GB VRAM) with a fixed random seed for reproducibility.

**Random seed protocol:** Unless otherwise specified, experiments are conducted with a single fixed random seed (seed = 11) for reproducibility. For selected ablation studies and cross-dataset robustness evaluations in Tables 4–8 and 13, results are averaged over three random seeds (11, 42, 123), and reported as Mean ± Standard Deviation (SD). Only mean values are reported for brevity in Tables 6–8. For all other tables (Tables 9–12, 14–16), results are obtained with a single fixed random seed (seed = 11).

#### Evaluation metrics.

Intersection over Union (IoU) is a fundamental metric in object detection that measures the overlap between a predicted bounding box and the ground truth bounding box:


$$\begin{array}{c} IoU=\frac{\left|B_{pred}\cap B_{gt}\right|}{\left|B_{pred}\cup B_{gt}\right|} \end{array}$$ (15)

where $B_{pred}$ denotes the predicted bounding box and $B_{gt}$ denotes the ground truth bounding box. A detection is considered correct if IoU≥threshold (commonly 0.5).

To measure the performance of our DAAF module and compare it with other methods, we adopt mAP@0.5 as the primary evaluation metric. mAP@0.5 calculates the average precision (AP) for each category when IoU=0.5. Besides the overall mAP, we report per-category APs to conduct a detailed analysis of the category-specific effectiveness of DAAF.


$$\begin{array}{cc} AP & =\int_{0}^{1}\frac{TP}{TP+FP}d\frac{TP}{TP+FN}\\ & =\int_{0}^{1}P(r)dr \end{array}$$ (16)

$$\begin{array}{c} mAP=\frac{1}{n}\sum_{i=1}^{n}{AP}_{i}=\frac{1}{n}\sum_{i=1}^{n}\int_{0}^{1}P_{i}(r)dr \end{array}$$ (17)

#### Baseline and comparison methods.

We compare DAAF with the methods in Table 3 to verify its effectiveness. Among them, the Add Fusion method fuses features via element-wise addition and contains no learnable parameters; the Concat Fusion method employs channel-wise concatenation followed by a 1×1 convolution for dimensionality reduction and includes learnable parameters. In the dual-branch fusion baseline, we mainly use Add Fusion (element-wise addition without parameters) as the main comparison baseline because it represents the most direct dual-branch fusion strategy and does not introduce additional parameters, providing a fair comparison. By comparing with Add Fusion, we can prove that the improvement of DAAF stems from the fusion strategy rather than simply increasing parameters. To ensure fairness in evaluation, all baseline models and ablation variants share identical training settings—differing solely in their feature fusion mechanisms.

**Table 3:** Baseline Methods and Comparative Methods.

Category	Method	UFDC	HCSS	SADPG
–	MobileNetV2	–	–	–
	MobileViT	–	–	–
Baseline	Concat Fusion	×	×	×
	Add Fusion	×	×	×
Ablation-1	UFDC Only	√	×	×
	HCSS Only	×	√	×
	SADPG Only	×	×	√
Ablation-2	UFDC + HCSS	√	√	×
	UFDC + SADPG	√	×	√
	HCSS + SADPG	×	√	√
DAAF	DAAF-Full	√	√	√

## Main experimental results

### Comparative evaluation of model performance

Table 4 provides a comprehensive performance comparison of DAAF with baseline methods across all three benchmark datasets.

**Table 4:** Multi-Dataset Performance Comparison (Mean±SD, mAP@0.5, %).

Method	NWPU	DOTA	DIOR	Average	vs Baseline
MobileNetV2	87.44 ± 1.27	68.96 ± 0.15	78.14 ± 1.12	78.18 ± 0.85	−2.49
MobileViT	80.97 ± 1.94	60.12 ± 2.73	86.46 ± 0.33	75.85 ± 1.67	−4.82
Add Fusion (baseline)	86.03 ± 1.90	69.26 ± 0.77	86.71 ± 1.86	80.67 ± 1.51	–
Concat Fusion	87.00 ± 0.77	69.98 ± 0.79	88.19 ± 0.16	81.72 ± 0.57	+1.05
**DAAF-Full (ours)**	**89.77 ± 0.81**	**70.18 ± 0.76**	**88.22 ± 0.42**	**82.72 ± 0.66**	**+2.05**
Enhancement vs. Baseline	+3.74	+0.92	+1.51	+2.05	–

**Note:** Results are averaged over three random seeds with standard deviation. Bold: best performance. vs Baseline: improvement relative to Add Fusion average performance. Enhancement vs. Baseline: DAAF-Full improvement over Add Fusion on each dataset.

Experimental results consistently validate the superior performance of DAAF across all three evaluation datasets. As shown in Table 4, the dual-branch fusion methods (Add and Concat) both achieved performance improvements compared to the single-branch methods, with average mAP reaching 80.67% and 81.72% respectively, surpassing MobileNetV2 (78.18%) and MobileViT (75.85%), which validates the complementarity of CNN-ViT features. DAAF-Full further consistently enhanced performance over the Add Fusion baseline, achieving a significant gain of +3.74% mAP on NWPU, with the average mAP increasing from 80.67% to 82.72% (+2.05%), proving the effectiveness of the DAAF fusion strategy in handling diverse remote sensing scenarios. The method’s robust performance gains observed across diverse datasets underscore its strong generalization capability.

#### Detailed analysis by category.

**Fig 4:**
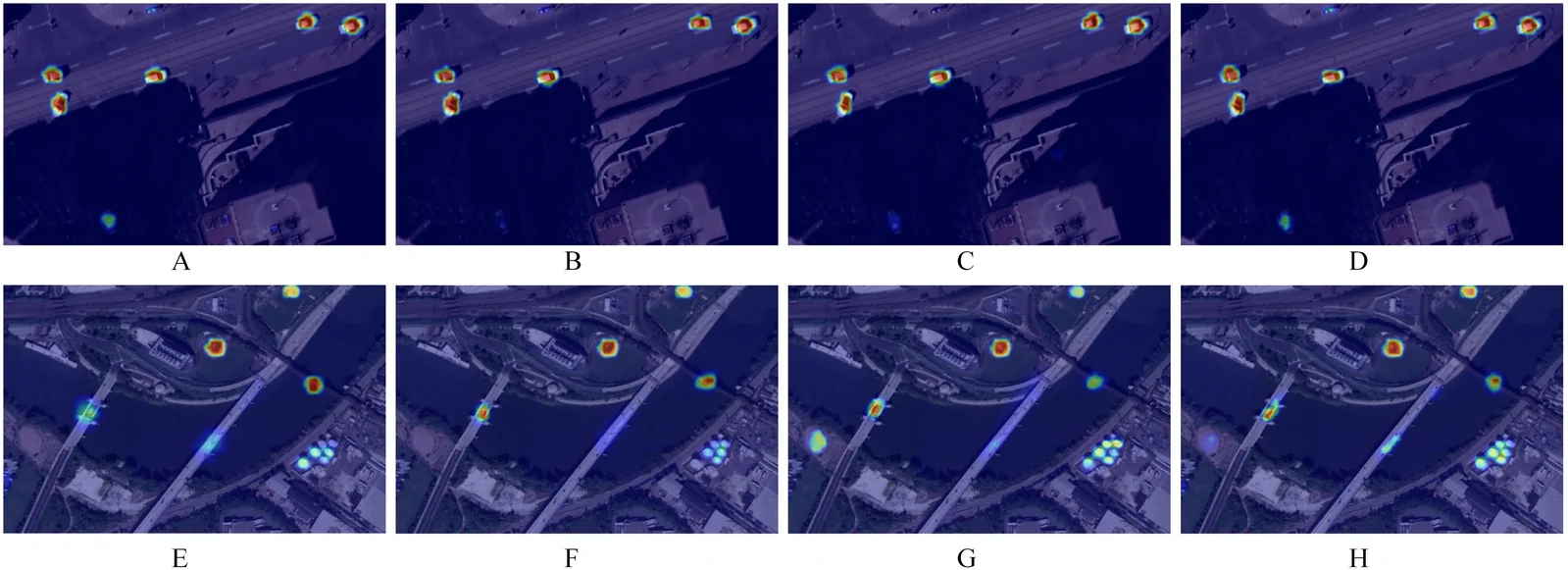
Feature activation heat maps for representative bridge and vehicle cases. (A–D) Feature activation heat maps for the bridge category generated by MobileViT, MobileNetV2, Add Fusion, and DAAF, respectively. (E–H) Corresponding feature activation heat maps for the vehicle category generated by MobileViT, MobileNetV2, Add Fusion, and DAAF, respectively. All feature activation heat maps were generated by the authors using their own experimental pipeline based on model inference results.

Table 5 reveals the per-category performance of DAAF-Full on the NWPU dataset. DAAF-Full achieves the most significant gains on BR (+17.36%), VH (+13.18%), and HB (+8.39%) over the Add Fusion baseline, demonstrating that the dual-attention fusion effectively captures fine-grained structural details of elongated and small-scale targets. These results validate that DAAF successfully integrates the local structural details of CNNs and the global spatial layout understanding of ViTs, proving the effectiveness of DAAF in leveraging CNN-ViT complementarity to handle complex remote sensing targets. Fig 4 presents the corresponding feature activation heat maps generated during model interpretation, further demonstrating that DAAF learns more discriminative feature representations.

**Table 5:** Per-category Detection AP on NWPU Dataset (Mean±SD, AP@0.5, %).

Category	MobileNetV2	MobileViT	Concat Fusion	Add Fusion	DAAF-Full (ours)	DAAF Gain
AP	**100 ± 0**	99.96 ± 0.03	**100 ± 0**	**100 ± 0**	**100 ± 0**	0
BD	97.56 ± 0.34	95.55 ± 0.72	**98.89 ± 0.69**	97.36 ± 1.14	96.9 ± 0.9	−0.46
BC	81.85 ± 5.25	57.48 ± 21.35	84.58 ± 4.97	**88.93 ± 6.38**	83.47 ± 4.16	−5.46
BR	84.61 ± 8.43	80.04 ± 13.51	76.41 ± 12.01	73.71 ± 7.56	**91.07 ± 6.44**	+17.36
GTF	**100 ± 0**	**100 ± 0**	99.74 ± 0.45	**100 ± 0**	**100 ± 0**	0
HB	85.92 ± 6.16	84.87 ± 1.49	88.05 ± 3.05	82.75 ± 3.37	**91.14 ± 4.42**	+8.39
SH	65 ± 5.46	54.18 ± 3.52	65.86 ± 6.48	63.69 ± 2.58	**67.04 ± 5.62**	+3.35
ST	96.19 ± 2.03	95.26 ± 0.82	**97.48 ± 1.83**	95.87 ± 2.66	96.41 ± 1.7	+0.54
TC	**90.66 ± 1.45**	86.61 ± 1.24	90.11 ± 1.05	89.89 ± 0.63	90.38 ± 0.51	+0.49
VH	72.57 ± 5.43	55.72 ± 3.63	68.92 ± 0.32	68.15 ± 4.87	**81.33 ± 2.24**	+13.18
**Overall mAP**	87.44 ± 1.27	80.97 ± 1.94	87.00 ± 0.77	86.03 ± 1.9	**89.77 ± 0.81**	+3.74

Per-category detection AP on the NWPU dataset averaged over three random seeds with standard deviation. DAAF gain: improvement of DAAF-Full over Add Fusion (baseline) on each category. **Bold**: best performance.

Tables 6 and 7 report the quantitative per-category detection performance on the DOTA and DIOR benchmarks, respectively. On DIOR, DAAF-Full achieves the highest overall mAP of 88.22% and ranks among the top two methods across 18 out of 20 categories, demonstrating consistent generalization across diverse object types.

**Table 6:** Per-category Detection AP on DOTA Dataset (Mean, AP@0.5, %).

Method	mAP	BD	BC	BR	GTF	HA	HC	LV
MobileViT	60.12	56.77	41.14	32.82	32.60	72.00	13.10	83.07
MobileNetV2	68.96	73.50	57.35	43.90	51.65	78.16	18.15	84.46
Add Fusion	69.26	71.83	**60.66**	44.15	48.53	77.79	21.50	84.44
Concat Fusion	69.98	**75.50**	59.37	45.27	52.41	78.49	21.54	**85.13**
DAAF-Full (ours)	**70.18**	72.56	59.87	**45.67**	**53.82**	**79.56**	**23.79**	84.82
**Method**	**PL**	**RA**	**SH**	**SV**	**SBF**	**ST**	**SP**	**TC**
MobileViT	94.71	36.97	91.66	70.04	44.27	81.42	59.96	91.35
MobileNetV2	96.19	57.45	93.46	71.61	64.75	87.36	63.57	**92.85**
Add Fusion	96.20	57.61	93.29	71.45	**64.79**	87.10	66.93	92.60
Concat Fusion	**96.54**	**59.96**	93.44	**73.56**	59.78	**87.56**	**68.66**	92.47
DAAF-Full (ours)	96.36	58.62	**93.54**	72.45	63.47	86.94	68.51	92.68

**Note:** Results are averaged over three random seeds. Only mean values are reported for brevity. **Bold**: best performance; Underline: second-best performance.

**Table 7:** Per-category Detection AP on DIOR Dataset (Mean, AP@0.5, %).

Method	mAP	C1	C2	C3	C4	C5	C6	C7	C8	C9	C10
MobileViT	86.46	94.59	90.28	95.44	93.26	59.98	90.93	78.79	96.48	91.64	91.18
MobileNetV2	78.14	90.66	84.06	92.25	90.42	49.01	87.08	64.56	89.89	81.58	82.18
Add Fusion	86.71	94.54	91.05	95.63	93.83	60.96	92.00	81.56	96.38	91.98	90.96
Concat Fusion	88.19	95.15	92.92	**96.21**	**94.47**	**64.35**	**93.60**	82.33	**96.87**	**93.78**	89.87
DAAF-Full (ours)	**88.22**	**95.19**	**93.65**	96.13	94.25	64.18	93.10	**83.95**	96.75	92.96	**91.71**
**Method**	**mAP**	**C11**	**C12**	**C13**	**C14**	**C15**	**C16**	**C17**	**C18**	**C19**	**C20**
MobileViT	86.46	91.68	74.38	76.29	95.15	94.24	90.01	97.63	69.12	70.84	87.20
MobileNetV2	78.14	84.55	60.16	65.50	86.99	93.04	77.25	94.91	58.07	50.13	80.45
Add Fusion	86.71	92.17	72.66	75.49	93.76	**94.74**	87.61	97.85	**78.62**	67.35	85.00
Concat Fusion	88.19	92.92	**75.98**	78.09	**95.96**	94.00	**91.62**	**98.27**	74.79	**74.50**	**88.12**
DAAF-Full (ours)	**88.22**	**93.17**	75.88	**78.47**	95.82	93.47	91.36	98.10	74.50	74.34	87.46

**Note:** Results are averaged over three random seeds. Only mean values are reported for brevity. **Bold**: best performance; Underline: second-best performance.

#### Cross-dataset consistency analysis.

To evaluate the cross-dataset generalization ability and the stability of the method’s applicability of DAAF, we analyzed the performance of the same categories on different datasets, as shown in Table 8.

**Table 8:** Cross-dataset Consistency Analysis (DAAF Gain, %).

Category	NWPU	DOTA	DIOR	Trend
Vehicle	+13.18	+1.00^*^	+6.99	Consistent ↑
Harbor	+8.39	+1.77	+3.22	Consistent ↑
Bridge	+17.36	+1.52	+3.22	Consistent ↑
Ship	+3.35	+0.25	+2.06	Consistent ↑
Airplane	0.00	+0.16	+0.65	Stable
Tennis Court	+0.49	+0.08	+0.25	Stable ↑
Ground Track Field	0.00	+5.29	+1.00	Stable ↑
Storage Tank	+0.54	−0.16	+3.75	Mixed
Baseball Diamond	−0.46	+0.73	+0.50	Mixed
Basketball Court	−5.46	−0.79	+0.42	Mixed ↓

DAAF Gain: improvement of DAAF-Full over Add Fusion (baseline) on each category. ^*^ DOTA does not have a Vehicle category; the value refers to Small Vehicle (SV).

As shown in Table 8, among the ten shared semantic categories across three datasets, seven show consistent or stable positive gains, demonstrating strong cross-dataset generalization. Vehicle, Harbor, Bridge, and Ship achieve consistent improvements across all datasets, with Bridge yielding the largest gain on NWPU (+17.36%). Near-saturated categories (Airplane, Tennis Court) show marginal but positive gains. Ground Track Field reaches saturation on NWPU yet yields meaningful gains on DOTA (+5.29%) and DIOR (+1.00%). The three mixed-trend categories (Storage Tank, Baseball Diamond, Basketball Court) exhibit occasional minor degradation attributable to inter-class similarity or dataset-specific distribution bias rather than systematic limitations of DAAF.

#### Comparison with the latest methods.

Tables 9–Table 10, 11 compares the performance of DAAF with the state-of-the-art (SOTA) methods on three datasets. DAAF achieves competitive performance on all datasets, demonstrating its robust generalization ability across different dataset characteristics. The above results confirm that the progressive fusion design of DAAF successfully integrates the complementarity of CNN and Transformer, achieving advanced detection performance.

**Table 9:** Performance of DAAF and Other Methods on Three Datasets (AP@0.5, %).

Method	NWPU	DOTA	DIOR
DAAF (ours)	89.77	**70.18**	**88.22**
MobileViT	81.11	56.92	86.41
MobileNetV2	86.13	68.85	79.43
YOLOv5 [55]	**92.04**	–	75.33
LO-Det [56]	–	66.17	65.85
RetinaNet [57]	–	67.45	65.70
Mask R-CNN [58]	83.97	–	60.61
Faster R-CNN [56,59]	80.90	–	63.10

Note: Results are obtained with a single fixed random seed. **Bold**: best performance; Underline: second-best performance.

**Table 10:** Performance of DAAF and Other Methods on the NWPU Dataset (AP@0.5, %).

NWPU	YOLOv5l [59]	YOLOv5s [59]	YOLOv7- tiny [59]	YOLOv4 [60]	Faster RCNN with FPN [61]	Faster R-CNN [61]	SSD [59]	R-P-Faster R-CNN [62]	YOLOv3 [63]	DAAF (ours)
mAP	**91.6**	88.3	87.2	86.7	82	80.9	78.4	76.5	76.4	89.77
AP	99.5	99.2	98.8	94.9	93.9	94.6	90.4	90.4	90.6	**100**
BD	98.9	**99.4**	99.2	98.3	95.7	95.5	89.9	89.9	94.8	96.9
BC	77	79.1	68.8	67.5	75.6	**89.7**	80.6	77.6	68.6	79.2
BR	78.7	82.1	72	**95.9**	66.8	57.5	76.7	68.2	58.1	91.07
GTF	99.2	97.6	98.3	99.3	88.5	92.4	98.3	87.7	92.1	**100**
HB	90.3	74	83.6	80.7	86.4	72.4	73.4	79.1	76.2	**91.14**
SH	**93.3**	84.1	89	78.6	72.3	82.3	60.9	75	63.1	67.04
ST	**99.3**	97.5	98.9	95.4	68.2	65.3	79.8	44.4	70.9	96.41
TC	87.9	89.5	85.8	88.2	**91.9**	81.9	82.6	79	83.8	90.38
VH	**91.4**	80.7	77.5	67.7	80.9	77.8	52.1	73.2	65.7	81.33

Note: Results are obtained with a single fixed random seed. **Bold**: best performance; Underline: second-best performance.

**Table 11:** Performance of DAAF and Other Methods on the DOTA Dataset (AP@0.5, %).

DOTA	RADet [64]	RetinaNet [57]	LO-Det [56]	Axis Learning [65]	YOLOv5s [66]	HyNet [67]	RRPN [68]	R^2^CNN [69]	DAAF (ours)
mAP	69.09	67.45	66.17	65.98	64.5	62	61.01	60.7	**70.18**
BD	76.99	**77.76**	66.14	77.15	67.6	58.7	71.2	65.7	72.56
BC	78.14	78.57	75.09	**79.07**	61.7	53.9	72.84	66.9	59.87
BR	**48.05**	47.47	31.32	38.59	41.4	43.7	31.66	35.3	45.67
GTF	65.83	59.07	55.96	61.15	51.6	54	59.3	**67.40**	53.82
HA	66.14	68.92	59.98	56.28	77.5	76.6	53.08	55.1	**79.56**
HC	**62.16**	38.22	48.42	36.05	46.1	40.3	53.58	48.2	23.79
LV	74.4	63.49	71.04	70.49	79.4	80.5	56.19	50.9	**84.82**
PL	79.45	88.28	89.22	79.53	88.3	86.7	88.52	81	**96.36**
RA	**64.63**	61.82	59.34	61.01	52.1	47.6	52.84	52.2	58.62
SH	68.86	77.69	84.27	76.3	80.7	87.8	57.25	55.8	**93.54**
SV	65.46	**73.83**	70.05	67.53	63.8	64.4	51.85	60	72.45
SBF	49.92	48.67	44.65	47.27	49.3	41.8	56.69	55.1	**63.47**
ST	74.97	65.87	81.28	83.53	62.5	60.3	67.38	72.4	**86.94**
SP	71.58	**71.59**	65.13	66.06	58.8	48.7	51.94	53.4	68.51
TC	89.7	90.43	90.74	89.66	90.8	85.1	90.81	90.7	**92.68**

Note: Results are obtained with a single fixed random seed. **Bold**: best performance; Underline: second-best performance.

These results demonstrate DAAF’s competitiveness with recent methods, DAAF’s gains are particularly pronounced on structurally complex categories (Bridge, Harbor, Vehicle), validating our hypothesis that CNN-Transformer complementarity benefits objects requiring both local details and global context. The consistent improvements across diverse dataset characteristics—from large-scale NWPU objects to small-scale DOTA objects to dense DIOR urban scenes—confirm DAAF’s robust generalization capability beyond dataset-specific tuning.

#### Analysis of inference speed.

We evaluated the inference efficiency on NVIDIA GPU, using per-image timing (each image is independently synchronized) to eliminate the impact of GPU pipeline effects (Table 12). Compared to Add Fusion baseline (53.45M parameters, 63.31ms), DAAF introduces only 206K additional parameters (0.37% increase) and 4.56ms latency (7.2% increase) while achieving 14.73 FPS, demonstrating a favorable accuracy-efficiency trade-off.

**Table 12:** Comparison of Inference Speed (NVIDIA GPU, Batch = 1, Input = 640×640).

Model	Params.(M)	Delay (ms)	FPS
MobileNetV2	7.73	58.46	17.10
MobileViT-S	47.57	54.13	18.48
Add Fusion	53.45	63.31	15.80
Concat Fusion	53.75	63.20	15.82
DAAF (ours)	53.65	67.87	14.73

#### Component contribution and interaction analysis.

We performed ablation analysis on the NWPU dataset (Tables 13 and 14) with three random seeds to ensure statistical reliability. As shown in Table 13, UFDC Only achieves 88.24 ± 1.36% mAP with only 1.3K parameters, demonstrating strong parameter efficiency on regular geometric objects. However, UFDC Only exhibits critical instability on structurally complex categories (BR: SD = 18.69%, CV = 23.01%, range = 34.93%), indicating that its competitive mean performance is unreliable and sensitive to random initialization.

**Table 13:** Ablation Experiment (NWPU Dataset, Multi-Seed Evaluation).

Configuration	Params. (K)	Mean±SD (%)	vs Add Fusion	Representative Category		
				BC	BR	VH
Add Fusion	0	86.03 ± 1.90	baseline	88.93 ± 6.38	73.71 ± 7.56	68.15 ± 4.87
UFDC Only	1.3	88.24 ± 1.36	+2.21	**98.75 ± 1.61**	81.19 ± 18.69^‡^	79.24 ± 2.51
HCSS Only	50.6	87.12 ± 0.63	+1.09	86.38 ± 1.30	87.36 ± 8.86	66.88 ± 4.18
SADPG Only	148	85.90 ± 0.79	−0.13	76.25 ± 4.41	75.45 ± 6.47	72.49 ± 4.71
UFDC + HCSS	51.9	87.83 ± 1.70	+1.80	83.87 ± 7.39	85.09 ± 10.24	73.19 ± 5.15
UFDC + SADPG	149	88.94 ± 0.97	+2.91	83.44 ± 4.29	84.34 ± 7.94	82.41 ± 2.87
HCSS + SADPG	199	88.22 ± 1.55	+2.19	84.93 ± 5.81	93.36 ± 8.39	69.29 ± 7.79
**DAAF-Full (ours)**	206	**89.77 ± 0.81**	**+3.74**	83.47 ± 4.16	**91.07 ± 6.44** ^§^	**81.33 ± 2.24** ^*^

^‡^BR UFDC Only: CV = 23.01%, range = 34.93% (high instability). ^§^ BR DAAF-Full: CV = 7.07%, range = 12.50% (2.90× variance reduction vs UFDC Only). * VH improvement statistically significant (*p* = 0.009).

**Table 14:** Efficiency Analysis (Selected Configurations).

Configuration	Delay (ms)	FPS
UFDC Only	62.89	15.90
UFDC + HCSS	33.69	29.68
UFDC + SADPG	63.80	15.67
DAAF-Full (ours)	67.87	14.73

UFDC Only recommended for parameter efficiency and regular objects; DAAF-Full recommended for variance control on challenging categories (Vehicle *p* = 0.009, Bridge 2.90× variance reduction).

DAAF-Full achieves 89.77 ± 0.81% with 206K parameters. While the + 1.53% mean improvement over UFDC Only is not statistically significant (*p* = 0.208), DAAF-Full provides three critical advantages that justify its deployment: (1) statistically significant improvement on Vehicle (81.33 ± 2.24% vs 79.24 ± 2.51%, *p* = 0.009), (2) 2.90× variance reduction on Bridge (SD: 18.69%→6.44%), and (3) 40% overall variance reduction (SD: 1.36%→0.81%). The 158× parameter increase is justified by the elimination of catastrophic variance and statistically reliable performance on challenging structural categories, which are essential for real-world remote sensing deployment.

Table 14 presents efficiency analysis. UFDC + HCSS achieves highest throughput (29.68 FPS), while DAAF-Full maintains 14.73 FPS. For scenarios prioritizing parameter efficiency and speed on regular objects, UFDC Only remains a viable choice. For applications requiring reliable performance across diverse and structurally complex categories, DAAF-Full is the recommended configuration.

#### Comparison of normalization methods.

Unless otherwise specified, analyses in this section are conducted with a single fixed random seed as they evaluate structural design choices whose relative conclusions are stable across seeds.

To verify the effectiveness of Instance Normalization in UFDC, we compared four normalization methods, keeping all configurations consistent (*r* = 16 for $P_{3}/P_{4}/P_{5}$ levels) to guarantee equitable evaluation. As shown in Table 15, Instance Norm achieved the best overall performance (89.64% mAP) and the lowest KL divergence (0.50), demonstrating its outstanding distribution alignment capability. Detailed per-category results are presented in Table 15, where Instance Norm excelled in key categories. All normalization methods reduced the KL divergence by 92–94%, confirming the effectiveness of normalization in feature alignment. These results confirm that Instance Norm is the optimal choice for UFDC.

**Table 15:** Comparison of UFDC Normalization Methods (NWPU Dataset, AP@0.5, %).

Configuration	mAP	AP	BD	BC	BR	GTF	HB	SH	ST	TC	VH	Before norm.	After norm.	Reduction rate
Non-normalized	86.44	**100**	98.03	**91.48**	83.67	**100**	86.02	62.16	92.45	90.19	60.42	–	–	–
Instance Norm	**89.64**	**100**	**98.42**	85.53	92.38	**100**	**91.43**	**67.21**	97.30	90.39	73.79	8.32	0.50	94.00%
Batch Norm	89.23	99.90	95.46	87.22	**94.81**	**100**	82.82	63.76	**98.87**	90.71	78.79	8.49	0.66	92.23%
Layer Norm	86.47	99.92	96.91	72.71	82.31	**100**	87.31	66.19	93.95	87.49	77.93	7.97	0.59	92.60%
Group Norm	86.33	**100**	98.09	87.74	66.12	**100**	77.30	64.48	97.77	**92.52**	**79.28**	7.34	0.58	92.10%

Note: Results are obtained with a single fixed random seed. **Bold**: best performance.

#### Channel reduction ratio configuration analysis.

In the multimodal feature pyramid fusion, features at different levels exhibit distinct characteristics: shallow features ($P_{3}$) contain rich spatial details, mid-level features ($P_{4}$) encode geometric semantics, and deep features ($P_{5}$) capture global context information. To fully exploit the feature modeling potential of each level, we explored an adaptive reduction ratio configuration strategy and evaluated its effectiveness (Table 16).

**Table 16:** Comparison of Compression Ratio Configurations (NWPU, AP@0.5, %).

Configuration (${𝐏}_{3}$, ${𝐏}_{4}$, ${𝐏}_{5}$)	mAP	VH	BC	BR	HB	Param.
(4,4,4)	89.21	68.16	81.89	81.96	81.69	218K
(8,8,8)	**90.16**	79.12	**92.58**	94.81	83.79	209K
(16,16,16)	89.79	74.30	83.24	95.71	90.51	203K
(4,8,16)	89.90	79.18	88.13	95.71	87.64	210K
(4,16,16)	90.02	**83.24**	79.20	**98.21**	**92.02**	206K

Note: VH = Vehicle, BC = Basketball Court, BR = Bridge, HB = Harbor. Results are obtained with a single fixed random seed. **Bold**: best performance.

Among the tested configurations, we selected (4,16,16) as the standard setting for DAAF despite the slightly higher overall mAP achieved by (8,8,8) (90.16% vs 90.02%). The category-level advantages of (4,16,16) on structurally complex and context-dependent targets—Vehicle (+4.12%), Bridge (+3.40%), and Harbor (+8.23%)—substantially outweigh the marginal 0.14% overall mAP difference. Therefore, our objective is not to maximize overall mAP alone, but to obtain a balanced configuration that maintains competitive overall accuracy while improving detection capability on challenging categories that better reflect practical remote sensing requirements.

This trade-off aligns well with practical remote sensing priorities, where detecting vehicles, bridges, and harbors—characterized by complex textures, variable scales, and cluttered backgrounds—typically demands higher accuracy than detecting objects with regular geometric patterns. Although the Basketball Court category exhibits a notable decrease (−13.38%), this category is characterized by relatively regular geometric structures and already achieves strong performance under multiple configurations. Moreover, (4,16,16) achieves better parameter efficiency, using 3K fewer parameters (206K vs 209K) than the uniform (8,8,8) configuration.

The underlying principle of this configuration is to match reduction ratios to the information density at each pyramid level. At the shallow $P_{3}$ level, a small reduction ratio (*r* = 4) preserves fine-grained details critical for complex object detection, while larger ratios (*r* = 16) at deeper levels ($P_{4}$ and $P_{5}$) improve computational efficiency without compromising performance on challenging categories. Since this comparison was conducted using a single fixed random seed, the results are interpreted as an exploratory configuration analysis rather than a definitive statistical comparison.

#### Verification of channel complementarity between CNN and ViT.

Channel attention analysis (Table 17) reveals the strong complementarity of CNN-ViT. At the shallow layer $P_{3}$, the two branches show a negative correlation (ρ = −0.256), and at the deeper layers $P_{4}-P_{5}$, the correlation is close to zero, indicating independent channel preferences. Crucially, the top 10 high-weight channels show zero overlap, confirming that the dual branches provide complementary information at the channel level.

**Table 17:** Channel Attention Analysis.

Fusion level	Number of channels	Relevance between two branches	Number of overlapping channels			
			Top10	Top20	Top50	Top100
fusion1($P_{3}$)	128	−0.256	0/10 (0%)	1/10 (10%)	17/50 (34%)	74/100 (74%)
fusion2($P_{4}$)	256	0.001	0/10 (0%)	1/10 (10%)	9/50 (18%)	41/100 (41%)
fusion3($P_{5}$)	256	0.057	0/10 (0%)	1/10 (10%)	11/50 (22%)	39/100 (39%)

Analysis is deterministic and independent of random seed selection.

**Fig 5:**
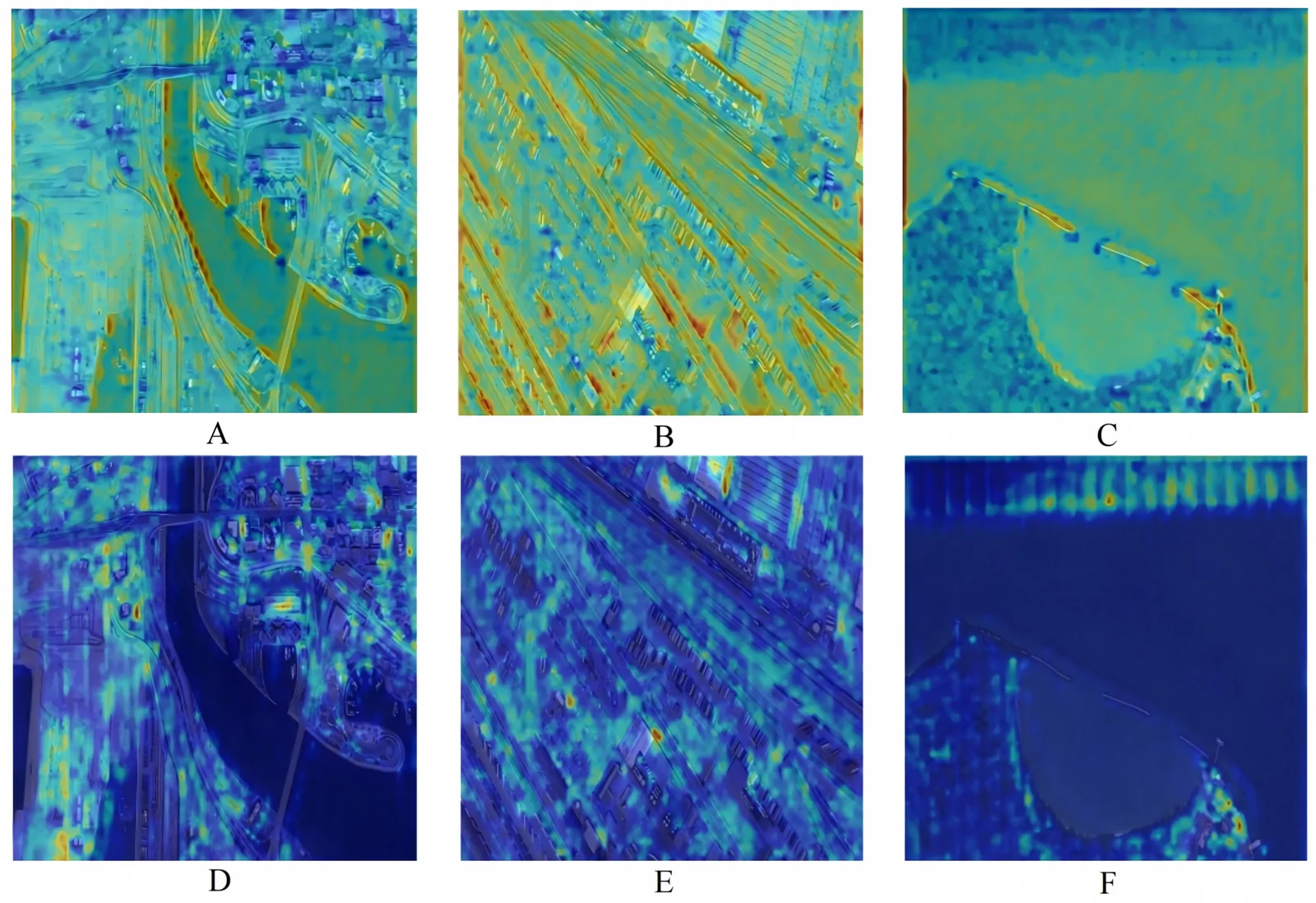
Grad-CAM visualization of CNN and ViT feature responses. Gradient-weighted Class Activation Mapping (Grad-CAM) visualizations show representative model-generated feature responses from the CNN and ViT branches. CNN responses primarily highlight local structural information, including edges and textures, whereas ViT responses emphasize broader semantic regions and global contextual information. All Grad-CAM visualizations were generated by the authors using their own experimental pipeline based on model-generated feature responses.

**Fig 6:**
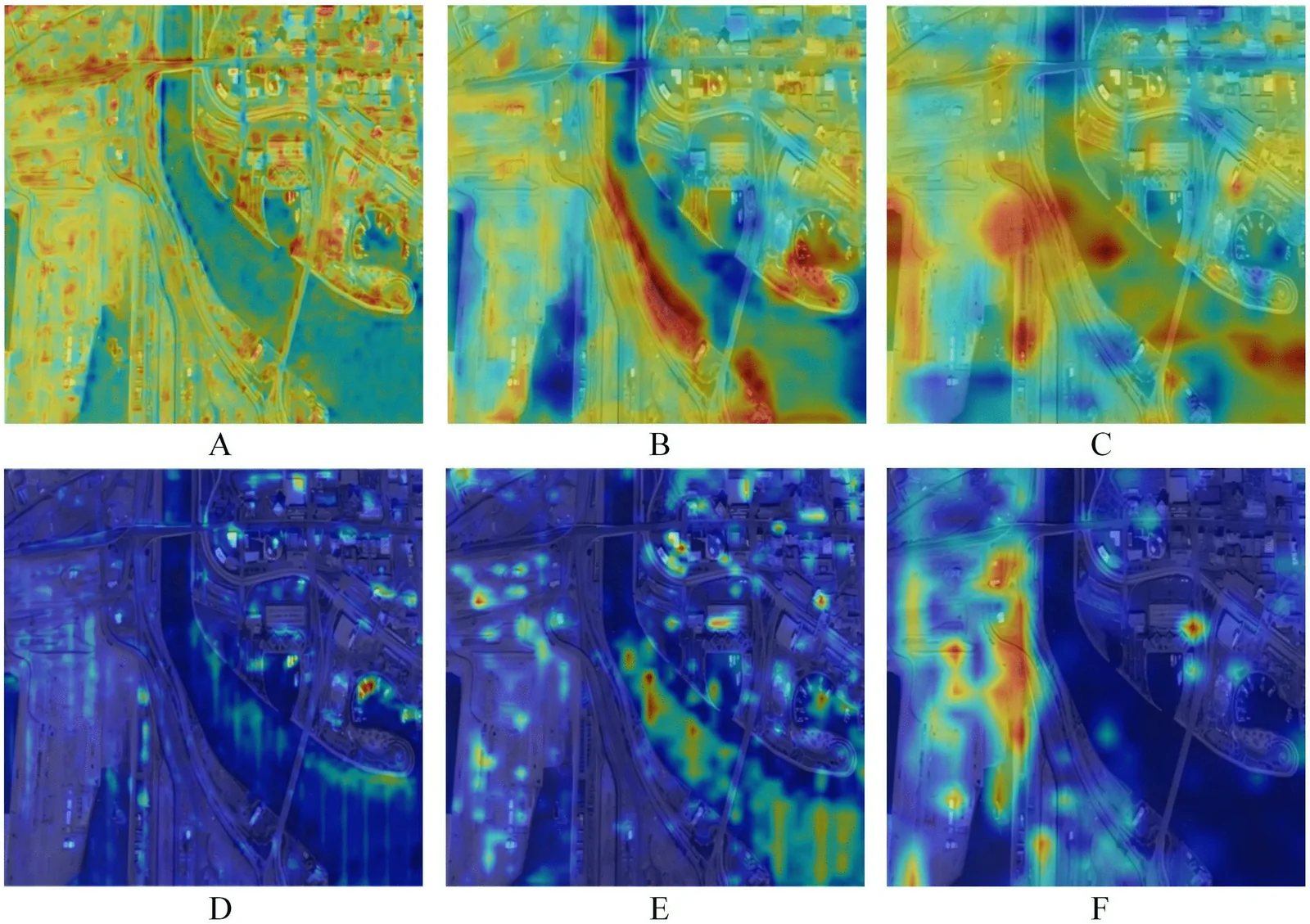
Grad-CAM visualization at different feature pyramid levels. (A–C) CNN feature responses at the $P_{3}$, $P_{4}$, and $P_{5}$ fusion levels, respectively. (D–F) ViT feature responses at the $P_{3}$, $P_{4}$, and $P_{5}$ fusion levels, respectively. The visualizations illustrate the evolution of local structural responses in the CNN branch and global semantic responses in the ViT branch across different feature pyramid levels. All visualizations were generated by the authors using their own experimental pipeline based on model-generated feature responses.

To further analyze feature complementarity, we employ Gradient-weighted Class Activation Mapping (Grad-CAM) to visualize the model-generated feature responses of different branches (Fig 5). The results show that the CNN branch accurately captures local details such as edges and textures, while the ViT branch grasps the global semantics of large regions. This complementarity across spatial, frequency, and functional aspects provides a possible explanation for the improved performance on challenging targets, including Bridge (+17.36%) and Vehicle (+13.18%). Multi-level analysis (Fig 6) further indicates that although the spatial resolution decreases from $P_{3}$ to $P_{5}$, the complementary pattern of edge responses from CNN and semantic responses from ViT remains consistent across the three levels, supporting the stability of the learned representations.

## Discussion

Our experimental results reveal several key findings regarding CNN-Transformer fusion for remote sensing object detection. Grad-CAM visualization (Fig 5) demonstrates clear complementarity: CNN branches activate on high-frequency features (edges, textures) while Transformer branches respond to low-frequency patterns (semantic regions, global context). Feature distribution misalignment (Table 15: KL divergence = 8.32) prevents simple fusion methods (Add Fusion: 80.67%, Concat Fusion: 81.72%) from fully exploiting this complementarity.

Ablation study (Table 13) confirms that UFDC is the primary performance driver (+2.21%), reducing KL divergence by 94%. The three-stage progressive design demonstrates clear additive benefits: UFDC alone achieves +2.21%, adding SADPG yields +2.91%, and DAAF-Full achieves +3.74%. Compared with UFDC Only, DAAF-Full provides an additional +1.53% mAP improvement, with substantially improved stability on Bridge (SD: 18.69→6.44) and statistically significant gains on Vehicle (*p* = 0.009). These results suggest UFDC is an efficient baseline, while DAAF-Full is preferable for challenging structural categories requiring robust cross-branch feature interaction.

Certain categories show partial configurations outperforming the full system: Basketball Court (−5.46pp vs Add Fusion) and Baseball Diamond (−0.46pp vs Add Fusion). This is due to Single-Branch Feature Sufficiency: CNN features alone optimally capture regular geometric patterns, while ViT adds limited value. Global optimization trade-offs in DAAF limit extreme single-branch configurations, but provide stability and higher gains for structurally complex categories (Bridge +17.36pp, Vehicle +13.18pp).

Cross-dataset analysis (Table 8) confirms that Vehicle, Harbor, Bridge, and Ship show consistent positive gains across NWPU, DOTA, and DIOR, indicating that DAAF’s advantages are systematic rather than dataset-specific.

Despite these advantages, two limitations remain: (1) uniform fusion weights across all categories limit performance on geometrically regular objects; (2) the 206K parameter overhead introduces moderate inference latency (14.73 FPS vs 15.90 FPS for UFDC Only). These insights directly motivate category-adaptive fusion(Future Directions), which dynamically adjusts fusion intensity based on category complexity: skipping full fusion for geometry-dominant objects and engaging three-stage fusion for context-dependent targets.

## Conclusions and future work

### Conclusions

This work investigates a fundamental challenge in CNN–Transformer fusion for remote sensing object detection: feature distribution heterogeneity and the resulting underutilization of cross-branch complementarity. To address this issue, we propose DAAF, a Dual-Stream Adaptive Attention Fusion framework consisting of three progressive stages: UFDC for feature distribution calibration, HCSS for complementary channel selection, and SADPG for discriminative information preservation.

Experimental results demonstrate that UFDC is the primary contributor to overall accuracy improvement, reducing the KL divergence from 8.32 to 0.50 (94% reduction) and providing a strong baseline gain of +2.21 percentage points over Add Fusion. Building upon this aligned feature space, HCSS and SADPG further improve fusion quality, enabling DAAF-Full to achieve an additional +1.53 percentage points mAP improvement over UFDC Only while substantially improving robustness on structurally complex categories. In particular, DAAF-Full reduces the Bridge standard deviation from 18.69 to 6.44 and achieves statistically significant gains on Vehicle (*p* = 0.009).

Across three benchmark datasets (NWPU VHR-10, DOTA v1.0, and DIOR), DAAF achieves improvements of +3.74, + 0.92, and +1.51 percentage points mAP over the Add Fusion baseline, respectively, with only 206K additional parameters (0.37% increase relative to the complete detector). Cross-dataset analysis further shows that seven of ten shared categories exhibit consistent positive trends, with particularly large gains on structurally complex targets such as Bridge and Vehicle. These results indicate that while UFDC provides an efficient lightweight baseline, the complete DAAF framework offers superior robustness and complementary feature utilization in challenging remote sensing scenarios.

### Future directions

The category-level analysis conducted in this study suggests two promising directions for future research.

(1) *Category-Adaptive Fusion.* Experimental results show that geometrically regular categories (e.g., Basketball Court and Baseball Diamond) can often be adequately represented by a single branch, whereas structurally complex categories (e.g., Bridge and Vehicle) benefit substantially from full CNN–Transformer fusion. This observation suggests that a category-adaptive fusion strategy could dynamically adjust fusion intensity according to target characteristics. Future work may leverage category priors derived from region proposals or intermediate predictions to selectively activate different fusion stages, thereby improving both accuracy and efficiency.
(2) *Multi-Modal Remote Sensing Fusion.* The proposed UFDC module is designed to address feature distribution heterogeneity and may naturally extend beyond CNN–Transformer fusion. Future research will investigate its application to multi-modal remote sensing scenarios, including SAR–optical, RGB–infrared, and hyperspectral–multispectral fusion, where distribution mismatch across sensing modalities remains a major challenge.
(3) *Efficiency-Oriented Deployment.* The results indicate that UFDC alone already achieves a favorable balance between accuracy and computational cost. Future work may therefore explore lightweight variants of DAAF, including conditional activation mechanisms and dynamic module selection, enabling deployment in resource-constrained remote sensing platforms while retaining the robustness advantages of the full framework.
